# Supplementary material for: CRISPR/Cas9 mediated gene editing in non-model nematode Panagrolaimus sp. PS1159
Source: Front Genome Ed. 2023 Feb 3;5:1078359. doi: 10.3389/fgeed.2023.1078359 (PMC9935820; doi:10.3389/fgeed.2023.1078359)
Supplement: Supplementary file 1 [file DataSheet1.pdf]

Supplementary Material

1 Supplementary Data

Supplementary video V1

2 Supplementary Figures and Tables

2.1 Supplementary Figures

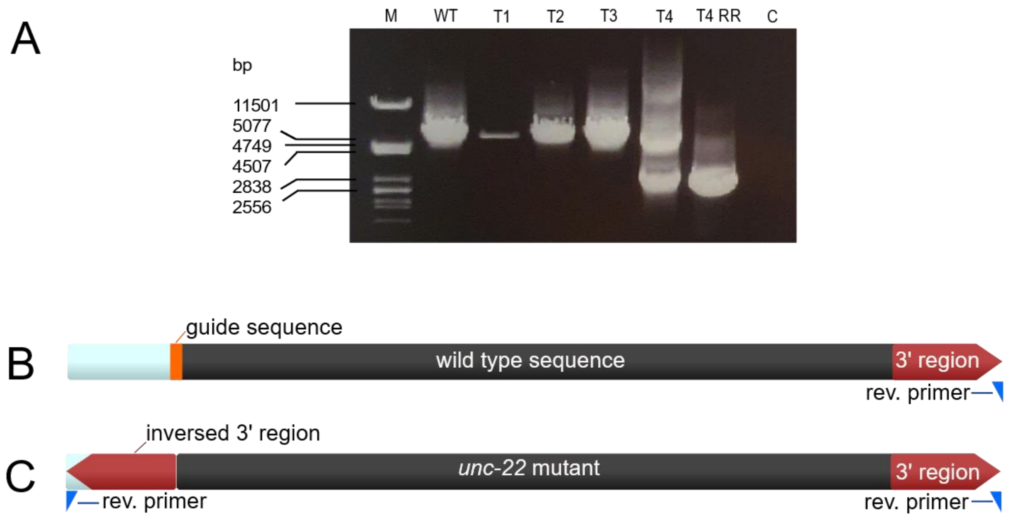

Supplementary Figure S1. (A) Agarose gel electrophoresis after PCR of 6000 bp region around the PS1159 *unc-22* target site with forward and reverse primers of mutant T1-T4. T4 RR= amplification of mutant T4 target region using reverse primers only. WT = wild type. C = Control. M = Marker Lambda *Pst*I digest **(B)** Schematic representation of wild type sequence around the *unc-22* target site **(C)** Schematic representation of *unc-22* mutant sequence (T4) with inversed 3' region and reverse primer site

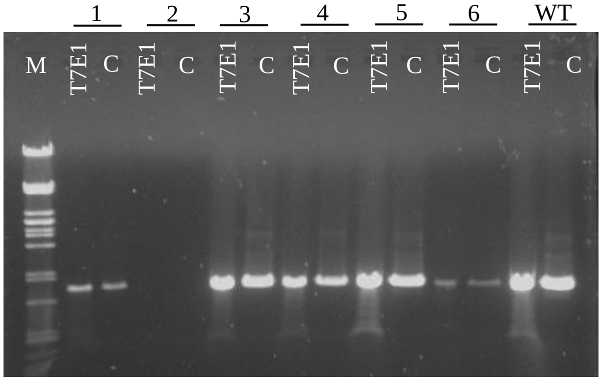

Supplementary Figure S2. Agarose gel electrophoresis after T7E1 assay of twitcher 1-6 (C=no T7 endonuclease, M=Marker Lambda *Pst*I)

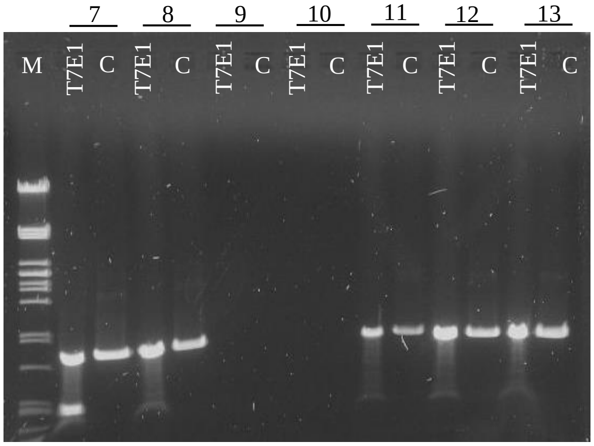

Supplementary Figure S3. Agarose gel electrophoresis after T7E1 assay of twitcher 7-13 (C=no T7 endonuclease, M=Marker Lambda *Pst*I); 7= T3 in figure 6

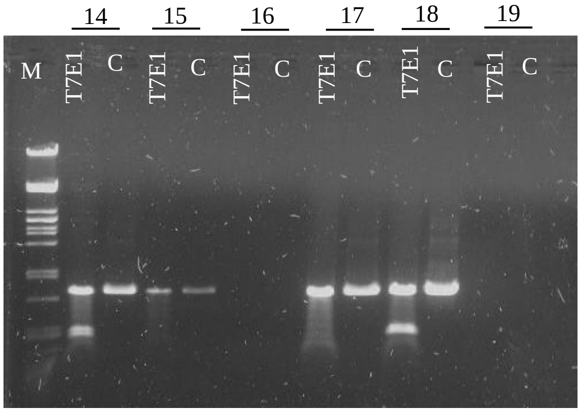

Supplementary Figure S4. Agarose gel electrophoresis after T7E1 assay of twitcher 14-19 (C=no T7 endonuclease, M=Marker Lambda *Pst*I); 14=T1, 18=T2 in Figure 6

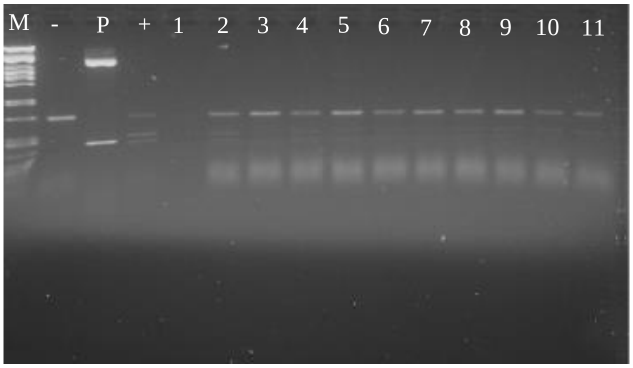

Supplementary Figure S5. Agarose gel electrophoresis after restriction digest of twitcher 1-11 (M=Marker Lambda *Pst*I, (-) negative control WT, (+) positive control, P=Plasmid with PstI restriction site)

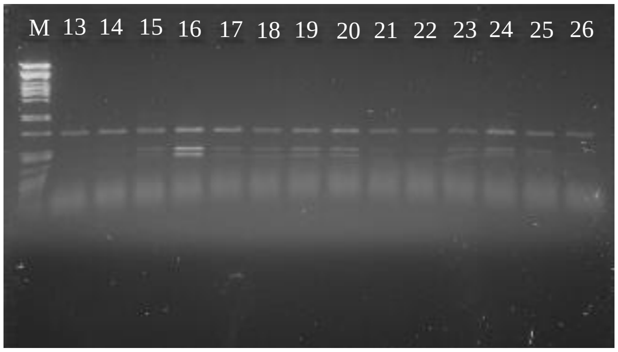

Supplementary Figure S6. Agarose gel electrophoresis after restriction digest of twitcher 13-26 (M=Marker Lambda *Pst*I, (-) negative control WT, (+) positive control, P=Plasmid with PstI restriction site); 13-17= T1-T5 in figure 5

2.2 Supplementary tables

Table S1: PNGG growth medium

**PNGG growth medium**
0.3 g Gelrite (Roth # 0039.1)
Ad 200 ml H2O
Autoclave and cool to room temperature
0.2 ml cholesterol (5mg/ml in 95% EtOH)

Table S2: Worm lysis buffer

**Worm lysis buffer**
10 mM Tris pH 8.0
50 mM KCl
2 mM MgCl2
0.5% NP40
0.5% Tween20
100 µg/ml Proteinase K

Table S3: M9 buffer

**M9 buffer**
3 g KH_2PO_4
6 g Na_2HPO_4
0.5 g NaCl
1 g NH_4Cl
Bring to 1 L with H_2O.

Table S4: Summary of control injections

	P0	Injected	Survived	Total F1	F1 with twitching phenotype
no crRNA	11	11	11	109	0
no Cas9	10	10	10	297	0
no injection	3	-	-	135	0
no injection	mixed	-	-	1300	0

Table S5: List of primers used for this study

Nematode	Name	Sequence (5'-3')
JU765	*unc-22* F1P	TCTGCAAAAAGATCTGACAGTGG
	unc-22 R1P	GATTGCATCAATTCTGCACTGG
	unc-22 F2	TGTGCCAAAATTGCATGAAGG
	unc-22 F1	GCGGCAATATCATGGAATCC
PS1159	*unc-22* R1	AGTTCTTTCAATCCTCGGAACC
	unc-22 SR2	GCTGTGTTCTGAATTTCCTGC
	unc-22 genF1	AACAAAGCTGGTGAAAGTGATCC
	unc-22 genR1	GGAGCACGTTTTGCTGGTCC
